# Identifying Influenza Viruses with Resequencing Microarrays

**DOI:** 10.3201/eid1204.051441

**Published:** 2006-04

**Authors:** Zheng Wang, Luke T. Daum, Gary J. Vora, David Metzgar, Elizabeth A. Walter, Linda C. Canas, Anthony P. Malanoski, Baochuan Lin, David A. Stenger

**Affiliations:** * Naval Research Laboratory, Washington, DC, USA;; † NOVA Research Inc., Alexandria, Virginia, USA;; ‡ Air Force Institute for Operational Health, Brooks City Base, San Antonio, Texas, USA;; § Naval Health Research Center, San Diego, California, USA;; ¶ Lackland Air Force Base, San Antonio, Texas, USA

**Keywords:** Influenza, virus, microarray, resequencing, random amplification, genotype, research

## Abstract

Identification of genetic variations of influenza viruses is essential for epidemic and pandemic outbreak surveillance and determination of vaccine strain selection. In this study, we combined a random amplification strategy with high-density resequencing microarray technology to demonstrate simultaneous detection and sequence-based typing of 25 geographically distributed human influenza virus strains collected in 2004 and 2005. In addition to identification, this method provided primary sequence information, which suggested that distinct lineages of influenza viruses co-circulated during the 2004–2005 season, and simultaneously identified and typed all component strains of the trivalent FluMist intranasal vaccine. The results demonstrate a novel, timely, and unbiased method for the molecular epidemiologic surveillance of influenza viruses.

## Abstract 2

Resequencing microarrays rapidly identify influenza viruses.

Influenza viruses are a major cause of respiratory infections in humans and result in substantial illness, death, and economic problems throughout the world. Along with regular seasonal epidemic outbreaks caused by common circulating strains, novel strains emerge sporadically because of reassortment in the segmented influenza RNA genome and have resulted in devastating influenza pandemics (*1**–**3*). Since mutations and reassortments are often determinants for infectious potential, antiviral drug susceptibility, and viral escape from vaccine-elicited immunity, continually surveying the genetic composition (i.e., primary sequence) of circulating and emerging variants is necessary. These needs have become increasingly relevant recently because the World Health Organization (WHO) has reported 85 human deaths caused by avian A/H5N1 influenza viruses throughout Asia since 2003 and raised concerns about the potential for another influenza pandemic (*4*).

Automated Sanger/electrophoresis-based sequencing technology has been used as the standard platform for DNA and genome sequencing. Although conventional sequencing produces accurate data, the requirement for knowledge of template sequences and the inability to quickly process multiple targets hinder its practical application in epidemiologic and diagnostic investigations. As an alterative, high-density oligonucleotide resequencing microarrays represent a promising new technology that has been used to rapidly and accurately identify nucleotide sequence variants (*5**–**7*) from viral, bacterial, and eukaryotic genomes (*8**–**13*). Use of resequencing microarrays to detect single nucleotide polymorphisms and generate primary sequences enables identification of genetic variants and provides valuable epidemiologic information that is critical for outbreak surveillance. In most cases, however, this technology has relied on specific amplification of a limited number of target sequences before hybridization, thus restricting throughput and limiting final identification to strains that retain primer-targeted sequences.

In an attempt to adapt resequencing microarray technology to surveillance and diagnostics, we developed the respiratory pathogen microarray (RPM) version 1 for detection and sequence typing of 20 common respiratory and 6 category A biothreat pathogens known to cause febrile respiratory illness (*14*). A large portion of RPM version 1 is focused on a subset of tiled sequences corresponding to partial fragments from the hemagglutinin (HA), neuraminidase (NA), and matrix (M) genes for detection of influenza A and B viruses. In this study, we demonstrate unbiased determination of viral subtype and lineage by generation of primary sequence using random nucleic acid amplification and resequencing microarray technology.

## Methods

### RPM Version 1 Design

Each tiled prototype sequence was selected to have an intermediate level of sequence homology across a group of microbial or viral strains, which allowed for efficient hybridization and unique identification of most or all subtypes of targeted pathogenic species. For each relevant base of a given prototype sequence, the array contains eight 25mer probes (4 sense and 4 antisense). Two of 8 probes represent perfect matches, while the others correspond to possible mismatches at the central (13th) position of the 25mers. The prototype regions targeting influenza viruses were composed of partial sequences from HA genes of influenza A virus subtypes (H1, H3, and H5) and influenza B virus, NA genes of influenza A virus subtypes (N1 and N2) and influenza B virus, and the M genes of influenza A virus (full-length M1 and partial M2) and influenza B virus (Table 1). Both HA and NA regions encompassed a sufficient number of polymorphic sites to define subtypes. These regions were combined with prototype sequences for 22 other pathogens and tiled on 12.8-μm chips (Affymetrix Inc., Santa Clara, CA, USA), which contain ≈240 K 25mer probes and have the capacity to resolve 30,000 nucleotides. The design and content of RPM version 1 array have been previously described (*14*).

**Table 1:** Influenza sequences tiled on the respiratory pathogen microarray version 1

Gene	Prototype	GenBank accession no.	Tiled region	Length (bp)
A/HA1	A/New Caledonia/20/99 (H1N1)	AJ344014	110–808	699
A/HA3	A/Denmark/59/03 (H3N2)	AY531939	120–913	794
A/HA5	A/Hong Kong/486/97 (H5N1)	AF102671	1106–1629	524
A/NA1	A/Chile/1/83 (H1N1)	X15281	4–1363	1,360
A/NA2	A/Panama/2007/99 (H3N2)	AJ457937	1–1446	1,446
A/M	A/NWS/33 (H1N1)	L25814	1–923	923
B/HA	B/Yamanashi/166/98	AF100355	269–952	684
B/NA	B/Yamagata/16/88	AY1139081	1–896	896
B/M	B/Yamagata/16/88	AF100378	1–362	362

### Sample Collection and Nucleic Acid Isolation

The influenza clinical specimens used in this study were collected through the Department of Defense Global Emerging Infections System during the 2004–2005 influenza season. Influenza throat swab specimens were collected in accordance with the case criteria previously described (*15*). Throat swabs were obtained within the first 72 h of the onset of symptoms, placed in viral transport medium (MicroTest M4, Remel Inc., Lenexa, KS, USA), and delivered by commercial carrier to the Air Force Institute for Operational Health in Brooks City Base, San Antonio, Texas, for culturing and molecular characterization. Specimens were passaged once through primary rhesus monkey kidney tissue culture (BioWhittaker, Walkersville, MD, USA). Cultures were tested for influenza A or B viruses by using the centrifugation-enhanced shell-vial technique with monoclonal antibody detection as previously described (*16*). Cultures testing positive for influenza A or B viruses were confirmed by using reverse transcription–polymerase chain reaction (RT-PCR) analysis with previously reported protocols (*16**,**17*). Total nucleic acids were extracted from 90-μL cultured samples or aliquots of live trivalent nasally administered influenza vaccine (FluMist 2004/05, MedImmune Inc., Gaithersburg, MD, USA) by using the MasterPure DNA purification kit (Epicentre Technologies, Madison, WI, USA) and dissolved in 30 μL of nuclease-free water.

### Random RT-PCR

Total RNA amplification from cultured samples using a random (RT-PCR) protocol was performed as previously described (*18*) with minor modifications. Briefly, 4 μL of total nucleic acids were reverse transcribed with 40 pmol of primer D (5´-GTTTCCCAGTAGGTCTCNNNNNNNNN-3´) and Superscript III reverse transcriptase (Invitrogen, Carlsbad, CA, USA) in a 20-μL volume at 42°C for 1 h, followed by heat denaturation at 70°C for 15 min. Aliquots (10 μL) of the RT reaction products were then amplified with the TaqPlus Long PCR System (Stratagene, La Jolla, CA, USA) for 35 cycles in a 50-μL volume consisting of 5 μL of 10× low salt buffer, 5 μL (25 mmol/L) MgCl_2_, 2 μL (10 mmol/L) dNTP mix, 1 μL primer E (100 mmol/L) (5´-GTTTCCCAGTAGGTCTC-3´), and 0.5 μL TaqPlus Long polymerase (5 U/μL). Each cycle consisted of 94°C for 30 s, 40°C for 30 s, 50°C for 30 s, and 72°C for 2 min. This was followed by a final extension at 72°C for 7 min. After amplification, the PCR products were purified with the QIAquick PCR Purification Kit (Qiagen, Valencia, CA, USA) and eluted in 45 μL of EB buffer (10 mmol/L Tris, pH 8.5).

### RPM Version 1 Hybridization and Processing

Purified DNA amplicons were adjusted to 2 μg in 35 μL of EB buffer, mixed with 15.1 μL of fragmentation cocktail buffer (5 μL NEB buffer 4, 5 μL 10 mmol/L Tris, pH 7.8, and 0.1 μL GeneChip fragmentation reagent [3 U/μL], Affymetrix Inc.), and incubated for 10 min at 37°C and 15 min at 95°C. The fragmented products were then biotin labeled with 1.5 μL of Biotin-N6-ddATP (PerkinElmer Life and Analytical Sciences, Boston, MA, USA) and 1 μL of terminal transferase (20 U/μL) (New England Biolabs, Beverly, MA, USA) for 45 min at 37°C and 15 min at 95°C. RPM version 1 arrays were prehybridized with 200 μL of prehybridization buffer (10 mmol/L Tris, pH 7.8, and 0.01% Tween 20) for 15 min at 45°C. After the prehybridization step, 167.5 μL of hybridization cocktail master mix (3 mol/L tetramethylammonium chloride, 10 mmol/L Tris, pH 7.8, 0.01% Tween 20, 0.5 mg/mL bovine serum albumin, 0.1 mg/mL herring sperm DNA [Promega, Madison, WI, USA], 50 pmol/L Oligo B2 [Affymetrix Inc.]) and biotin-labeled DNA fragments were heated for 5 min at 95°C, equilibrated for 5 min at 45°C, and added to RPM version 1. All hybridizations were incubated for 16 h at 45°C in the GeneChip hybridization oven 640 at 60 revolutions per minute. The microarrays were then washed and stained with the GeneChip Fluidics Station 450 and scanned with the GeneChip Scanner 300 according to the GeneChip CustomSeq array protocol.

The hybridization intensities were analyzed with the GeneChip operating software to generate raw image files (.DAT) and simplified image files (.CEL) with intensities assigned to each of the corresponding probe positions. GeneChip DNA analysis software version 3.0 (GDAS), which implements the ABACUS algorithm (*7*), was used to produce an estimate of corrected base calls file (.CHP). Base calls generated from each tiled region of the array were then exported from GDAS as Federal Acquisition Streamlining Act (FASTA)–formatted sequences.

### DNA Sequencing

Automated DNA sequencing was performed as previously described (*17*). HA nucleotide sequences for influenza strains used in this study are available at GenBank (accession nos. DQ265706–DG265730). The nucleotide sequences of primers used for amplification and sequencing are available upon request.

### Sequence Analysis

DNA sequences generated from RPM version 1 were searched against the Influenza Sequence Database (http://www.flu.lanl.gov/) (*19*) by using the BLAST algorithm (*20*). Advanced options for blast*n* search were set as follows: –W (word size) 7, –r (reward for a nucleotide match) 1, –q (penalty for a nucleotide mismatch) –1. These parameters were chosen to maximize sensitivity and allow sequences with as many as 50% ambiguous calls to still produce full-length searches. Sequence alignments were performed with the ClustalX program (ftp://ftp-igbmc.u-strasbg.fr/pub/ClustalX/).

## Results

### Microarray Hybridization

**Figure 1:**
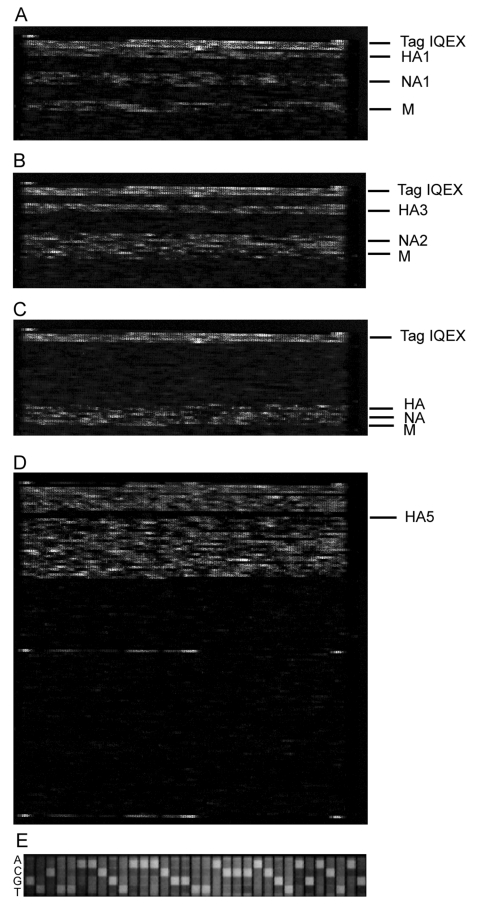
Hybridization images of the respiratory pathogen microarray (RPM) version 1 prototype regions for 3 influenza virus isolates and trivalent FluMist vaccine. A) A/H1N1, B) A/H3N2, C) influenza B, and D) trivalent FluMist vaccine. In A, B, and C, only the influenza-specific tiled prototype regions of RPM version 1 are shown. Hybridization-positive identifications are shown on the right. In D, the image of the entire RPM version when hybridized with FluMist vaccine is shown. The single influenza prototype region that was hybridization negative is denoted on the right. E) Magnification of a portion of profile B showing an example of the primary sequence data generated by the hybridization of randomly amplified targets to the RPM version 1 HA3 probe set. The primary sequence generated can be read from left to right. HA, hemagglutinin; NA, neuraminidase; IQEX, internal positive hybridization control (Affymetrix); M, matrix.

To assess the performance of RPM version 1 with a real-world clinical isolate set, we tested 25 cultured strains collected from 4 continents during the 2004–2005 influenza season and previously diagnosed by culture and RT-PCR as influenza. One influenza subtype was identified in each tested sample based on the RPM version 1 hybridization profiles and sequence reads shown in Figure 1A–C and E. DNA fragments of HA1, NA1, and M genes randomly amplified from an H1N1 isolate specifically hybridized to their corresponding prototype regions on RPM version 1 (Figure 1A). Prototype regions of 1 influenza subtype exhibited no interference from other subtypes (Figure 1A–C), and prototype regions of other pathogens on RPM version 1 showed no cross-hybridization with any influenza virus segments (Figure 1D). Of the 25 isolates tested, we identified 12 A/H3N2, 12 influenza B, and 1 A/H1N1 (Table 2). The A/H1N1 and A/H3N2 subtypes effectively hybridized to the same prototype M sequence (derived from the A/NWS/33 H1N1 strain), confirming that M genes are conserved among different H/N subtypes of influenza A to allow the universal identification of influenza A subtypes with a single tiled prototype region. A computational hybridization simulation model we developed confirms this suggestion (A. Malanoski, unpub. data). Aside from the highly conserved matrix region, no cross-hybridization was observed between subtypes, which suggests that the more variable HA and NA tiles are subtype specific. The GDAS generated DNA sequences from 3 genes (HA, NA, and M) from each sample with 42%–92% of the prototype tiled sequences, resulting in unambiguous calls by the microarray (Table 2). To demonstrate the accuracy of microarray resequencing reads, the HA genes from all 25 samples were amplified by a specifically primed RT-PCR and subjected to conventional sequencing. The sequences produced by random amplification and RPM version 1 were identical to those identified by the conventional sequencing method with the exception of ambiguous base calls (Ns). That is, in cases where both methods assigned a base identity at a particular sequence position, those assignments were always identical (data not shown).

**Table 2:** Influenza strain identification with respiratory pathogen microarray (RPM) version 1 versus conventional sequencing*

Sample name	Base call rate† (%)						
	HA	NA	M	Strain identification from HA	GenBank accession no.	M1‡	M2§
A/Colorado/360/05	84.4	72.7	63.2	A/Nepal/1679/2004 (H3N2)	AY945284	0	8
A/Qater/2039/05	88.4	74.0	68.5	A/Nepal/1727/2004 (H3N2)	AY945272	0	8
A/Guam/362/05	87.3	75.8	63.3	A/Nepal/1679/2004 (H3N2)	AY945264	2	10
A/Italy/384/05	83.3	69.6	63.5	/Nepal/1727/2004 (H3N2)	AY945272	2	9
A/Turkey/2108/05	77.9	67.6	59.2	A/Nepal/1664/2004 (H3N2)	AY945265	2	12
A/Korea/298/05	82.7	70.5	61.7	A/Nepal/1727/2004 (H3N2)	AY945273	4	11
A/Japan/1337/05	87.5	76.7	67.4	A/Malaysia/2256/2004 (H3N2)	ISDN110616	4	14
A/Japan/1383/05	92.1	84.9	74.8	A/Malaysia/2256/2004 (H3N2)	ISDN110616	4	14
A/Ecuador/1968/04	87.7	75.2	58.3	A/New York/17/2003 (H3N2)	CY001053	0	4
A/Iraq/34/05	84.4	72.7	65.6	A/Christchurch/178/2004 (H3N2)	ISDN110530	1	9
A/Peru/166/05	86.9	79.0	65.9	A/Macau/103/2004 (H3N2)	ISDN64772	6	10
A/New York/2782/04	82.7	68.0	63.1	A/New York/391/2005 (H3N2)	CY002056	1	9
A/England/400/05	88.3	55.3	61.1	A/New York/227/2003 (H1N1)	CY002536	1	10
B/Peru/1324/04	75.2	83.4	89.4	B/Milano/66/04	AJ842082	1	25
B/Peru/1364/04	71.1	74.5	77.5	B/Milano/66/04	AJ842082	1	25
B/Colorado/2597/04	81.1	84.3	85.8	B/Texas/3/2002	AY139049	4	27
B/Japan/1905/05	76.2	76.5	76.6	B/Texas/3/2002	AY139049	2	25
B/Japan/1224/05	80.0	78.2	83.7	B/Texas/3/2002	AY139049	2	25
B/Alaska/1777/05	75.0	75.9	78.1	B/Texas/3/2002	AY139049	4	27
B/England/1716/05	80.5	81.4	85.2	B/Texas/3/2002	AY139049	2	25
B/England/2054 /05	81.1	80.1	78.7	B/Texas/3/2002	AY139049	1	24
B/Hawaii/1990/04	51.7	82.9	83.7	B/Tehran/80/02¶	AJ784042	4	68
B/Hawaii/1993/04	47.4	79.7	83.7	B/Tehran/80/02¶	AJ784042	4	69
B/Arizona/148/04	42.4	78.2	82.5	B/Tehran/80/02¶	AJ784042	6	69
B/Arizona/146/04	49.1	79.1	86.7	B/Tehran/80/02¶	AJ784042	6	69

*HA, hemagglutinin; NA, neuraminidase; M, matrix. †No. of base calls generated from the RPM version 1 divided by the length of the tiled probe sequence. ‡No. of mismatches between the actual sequence and the sequence of the top BLAST search hit. §No. of mismatches between the actual sequence and the tiled prototype probe sequence. ¶Influenza B group 2 isolates were identified as B/New York/1/2002 strain (accession no. AF532565) by the conventional sequencing method.

### Sequence Analysis and Strain Identification

Microarray resequencing data and conventional sequencing data were searched by using the Influenza Sequence Database with the BLAST algorithm. Results for the highest bit scores were taken as strain identifications and are shown in Table 2.

### Influenza A

Based on sequences of HA genes, which are routinely used for genetic and antigenic characterization, microarray strain identifications of all 13 influenza A isolates correlated with identifications from the conventional sequencing method. Although A/H3N2 isolates were sometimes matched with different specific strain sequences from the Influenza Sequence Database based on the top BLAST hits for each isolate, all were redundant representatives of the same A/Fujian/411/02 lineage identified by conventional sequencing. These results indicate that ambiguous calls (Ns) did not affect the accuracy of BLAST identification. At most, only 6 mismatches occurred between the actual sequence of each isolate and sequence of its top BLAST search hit (Table 2, column M1).

**Figure 2:**
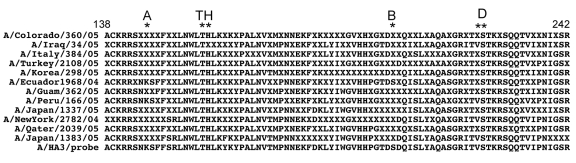
Alignment of hemagglutinin peptide sequences containing an influenza A/H3N2 prototype and the translated sequences from 12 A/H3N2 isolates generated from respiratory pathogen microarray version 1. A, antibody-binding site; TH, antibody-binding site Fujian-like lineage amino acid substitutions threonine and histidine; B, antibody-binding site; D, antibody-binding site. Asterisks indicate conserved amino acids.

Alignment of the HA peptide sequences translated from RPM version 1–obtained DNA sequences for 12 A/H3N2 isolates (Figure 2) showed that they all shared signature Fujian-like lineage amino acid substitutions (threonine and histidine) at positions 155 and 156 (*17*). Serine (position 227), which is located within antibody binding site D, was also conserved in these isolates, distinguishing them from the A/California/7/04 strain, which has proline at this position (*17*). In addition, isolate A/Ecuador/1968/04 shared similar amino acids with those observed in the A/Fujian/411/02 strain at antigenic sites A (lysine, position 145) and B (serine, position 189). Because of a more limited collection of NA and M gene sequences in the Influenza Sequence Database, strain identifications based on these 2 genes could only place them into clade A strains of H3N2 influenza A viruses sampled from New York State, which caused the A/Fujian/411/2002-like epidemic of the 2003–2004 influenza season (data not shown) (*21*). Although the only tiled M sequence was adopted from an A/H1N1 strain (A/NW/33), M results generated from the H3N2 isolates were still clearly identifiable as belonging to the A/H3N2 subtype and more specifically to the Fujian-like strain. The A/England/400/05 isolate was the only isolate appropriately identified as A/H1N1, and all 3 sequences (HA, NA, and M) generated from RPM version 1 and conventional sequencing for this isolate matched A/New York/227/2003 (H1N1). This is an A/New Caledonia/20/99-like strain that has been consistently circulating globally since 1999 (*16*).

### Influenza B

The 12 influenza B isolates were classified as belonging to 2 distinct subgroups based on BLAST searches of 3 genes generated from RPM version 1 analysis. The top BLAST hits for the RPM-obtained sequences of the HA gene identified subgroup 1 isolates as either B/Milano/66/04 or B/Texas/3/2002, both of which are B/Shanghai/361/2002-like strains and belong to the B/Yamagata/16/88 lineage. BLAST queries of conventional sequencing data yielded similar identifications for these isolates. Subgroup 2 isolates were identified as B/Tehran/80/02 by microarray and as B/New York/1/2002 by conventional sequencing. The query results of both methods were similar (different identification can be attributed to ambiguous base calls), and all isolates were members of the B/Victoria/2/87 lineage. This lineage is not covered by the 2004–2005 influenza B vaccine (L. Daum, pers. comm.). These results correspond to a Centers for Disease Control and Prevention (Atlanta, GA, USA) influenza activity report documenting that both of the identified influenza B lineages were reported worldwide and that the Yamagata lineage viruses predominated in the 2004–2005 influenza season (*22*).

### Genotyping

**Figure 3:**
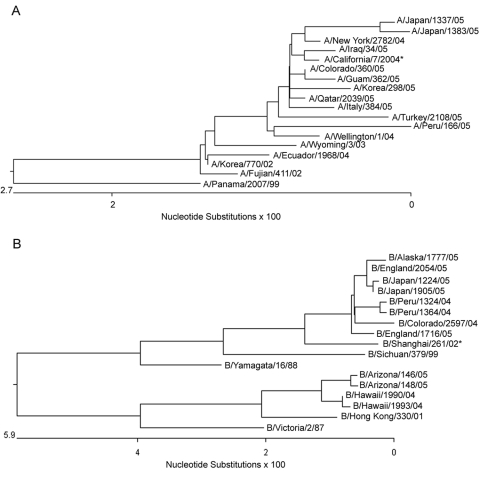
Unrooted phylogenetic analysis of the hemagglutinin 1 (HA1) gene of A) 11 influenza A/H3N2 isolates and B) 12 influenza B isolates compared with vaccine and reference strains. All clinical isolates are available from GenBank under accession nos. DQ265706–DG265730. *denotes the 2005–2006 influenza A/H3N2 and influenza B vaccine strains.

RPM version 1 can differentiate a broad number of variants based on a single-tiled "prototype" probe region without relying on predetermined hybridization patterns (*9*). A number of nucleotide mismatches that distinguished tested isolates from tiled prototype probe sequences were identified in each sample (Table 2, column M2). Some were unique with respect to existing influenza database-recorded sequences. All of these polymorphisms were verified by conventional sequencing (Table 3). Analysis of HA sequences generated from 12 A/H3N2 isolates by RPM version 1 showed that 4 of these nucleotide variations are common to 11 of the samples, excluding the outlying A/Ecuador/1968/04 isolate. Two of these common base substitutions, 313 G→A and 352 A→C, are at the third nucleotide of their respective codons and represent synonymous mutations. Such mutations do not code for amino acid changes and are usually selectively neutral and much more likely to be shared by common ancestry than by parallel evolution. These facts strongly support phylogenetic grouping of these 11 strains (Figure 3). In contrast, 393 A→T and 483 G→A are nonsynonymous mutations and code for critical amino acid changes. Analysis of conventional sequencing data confirmed that these 2 positions are in the antigenic site B and that the affected amino acids were changed from tyrosine to phenylalanine and from serine to asparagine, respectively. These 2 substitutions are both characteristic features of the A/California/7/04 strain that distinguish this group at both sequence and antigenic levels from other Fujian-like strains. The identified polymorphisms show that 11 of the 12 A/H3N2 isolates, although collected from 4 continents, are members of the same A/California/7/04 lineage, while the lone outlier, A/Ecuador/1968/04, is clearly identified as a member of the older A/Fujian/411/02 lineage. These observations demonstrate that RPM version 1 data can be effectively used for molecular epidemiologic tracking.

**Table 3:** Nucleotide differences among hemagglutinin (HA) influenza virus genes identified by respiratory pathogen microarray (RPM) version 1

Position†	TN‡	Mismatched nucleotides*											
		1	2	3	4	5	6	7	8	9	10	11	12
25	T												C
46	T		A										
61	A					T							
62§	G								A				
88	G										T		
189	G						A						
208	T										C		
233§	G												**A**
244	G												A
251§	G						A	A					
262	C								T				
274	G										A		
293§	A		G										
299	G	A	A	A	A	A	A	A	A	A	A	A	A
313§	G	**A**		**A**	**A**	**A**	**A**	**A**	**A**	**A**	**A**	**A**	**A**
351§	A						G	G		G			
352	A	**C**		**C**	**C**	**C**	C	C	C	C		**C**	**C**
385	C				T								
393§	A	**T**		**T**	**T**	**T**	**T**	**T**	**T**	**T**	**T**	**T**	**T**
407	T					C							
429§	A				G								
434§	A						**G**	**G**					
446§	T												C
466	C								T				
469	C								T				
473§	G									A			
478	T						A	A					
479§	G						T	T					
483§	G	**A**		**A**	**A**	**A**	A	A	**A**	**A**	**A**	**A**	**A**
493	C			T									
511	A	**T**		**T**									
559	C										T		
564§	A												G
584§	G												A
571	A						G	G					
593§	G	A		A	**A**	**A**	A	A	A	A		A	
596§	T	C		C	C	C	C	C	C	C	C	C	C
602§	A		C										
646	T										C		
652	T			A									
698§	C											A	
734§	C							T					

*Mismatched nucleotides obtained from comparison between the tiled probe sequence and the conventional sequence. Mismatched nucleotides identified by the RPM version 1 are shown in **boldface**. 1–12 represent 12 influenza A/H3N2 isolates. 1, A/Colorado/360/05; 2, A/Ecuador/1968/04; 3, A/Guam/362/05; 4, A/Iraq/34/05; 5, A/Italy/384/05; 6, A/Japan/1337/05; 7, A/Japan/1383/05; 8, A/Korea/298/05; 9, A/New York/278/04; 10, A/Peru/166/05; 11, A/Qatar/2039/05; 12, A/Turkey/2108/05. †HA3 tiled prototype sequence nucleotide position. ‡Tiled nucleotide. §Potential nonsynonymous mutation position.

Nearly every isolate was shown to have unique base mutations, many of which resulted in amino acid substitutions. Identification of these mutations reaffirms common knowledge that genetic drift is a frequent event during circulation of influenza viruses and that the RPM version 1 gene chip is an effective tool for tracking unique genetic changes within influenza strains.

### Detection of Multiple Targets

To test the capability of RPM version 1 to detect multiple pathogens with the random amplification protocol, we analyzed total nucleic acid isolated from trivalent FluMist intranasal vaccine. Figure 1D shows that 8 tiled influenza sequences on RPM version 1 were strongly hybridized by randomly amplified FluMist nucleic acids, and the resulting sequence data confirmed that FluMist includes immunogenic surface protein (HA and NA) genes from influenza H1N1, H3N2, and influenza B strains. Sequence analysis showed that these antigen-encoding genes matched those of 3 wild-type influenza strains recommended by WHO for making vaccine for the 2004–2005 season (Table 4). Two types of M genes from FluMist were identified by RPM version 1 as those in the cold-adapted Ann Arbor strains of influenza A and B, both of which are essential components in the cold-adapted master donor virus vaccine strain (*23*).

**Table 4:** Strain component analysis of the FluMist vaccine with respiratory pathogen microarray (RPM) version 1*

Segment	Base call rate† (%)	Strain identification	GenBank accession no.
A/HA1	86.8	A/New Caledonia/20/99	AJ344014
A/NA1	65.6	A/New Caledonia/20/99	AJ518092
A/HA3	86.5	A/Wyoming/3/03	AY531033
A/NA2	78.2	A/Wyoming/3/03	AY531034
A/M	75.9	A/Ann Arbor/6/60	M23978
B/HA	77.1	B/Jilin/20/2003	ISDN40908
B/NA	83.5	B/Yamagata/1246/2003‡	AB120256
B/M	78.4	B/Ann Arbor/1/66	M20175

*HA, hemagglutinin; NA, neuramindase; M, matrix. †No. of base calls generated from the RPM version 1 divided by the length of the tiled probe sequence. ‡The NA sequence of B/Jilin/20/2003 strain was not available in the influenza database.

## Discussion

Because of the relative ease of transmission of respiratory pathogens, tremendous pressure exists to develop rapid and sensitive tools to identify them. The surveillance of influenza virus outbreaks requires identification not only on the species level but also on the subtype or strain level. Current molecular methods, such as PCR and multiplex PCR, have dramatically improved detection sensitivities and efficiency compared with culture and serologic methods (*24*). However, they require multiple diagnostic tests to discriminate between organisms at multiple phylogenetic levels and are inherently limited in scope and resolution (i.e., increases in resolution necessitate corresponding decreases in scope). Furthermore, these tests rely on the conservation of primer-targeted sequences and as such can be rendered completely ineffective by as little as a single base mutation.

Currently, most microarrays used for microbial detection are spotted arrays that use redundant oligonucleotides as independent probes. For these methods, 2 types of probe targets are usually considered. The first are conserved gene sequences such as 16S rRNA and gyrase (*25**,**26*), which are chosen for identification at the genus or family levels. The second are relatively unique sequences such as virulence factor genes and antigenic determinant genes (*27**,**28*), which are used for species or serotype identification. In this way, pathogen recognition by microarray becomes as reliant on specific hybridization patterns as PCR is on primer-target conservation. Thus, a microarray is only able to resolve identity to the level of divergence represented by the diversity of probes present on the array. With resequencing arrays such as RPM version 1, multiple contiguous sequences (range 100 bp to 2 kb) containing both conserved and unique target genes from each species or subtype can be selected as prototype regions, and every nucleotide from the hybridized target regions can be potentially read as an independent data point using resequencing algorithms (*5*). The key advantage of the resequencing array is that it does not require a specific match between the analyzed sample and the probe, and mismatches actually add value because they can be identified and used as strain-specific markers.

Since the antigen-encoding HA and NA genes are highly variable between different subtypes, sequences specific for HA1, HA3, HA5, NA1, and NA2 were all tiled on RPM version 1 independently so that influenza A H3N2, H1N1, and H5N1 viruses could be identified and resequenced. Further analysis of the generated sequences showed variations between target and prototype sequences and accurately identified tested isolates at the strain level and as members of recognized circulating variants (Table 2). With its capability to identify strains, the resequencing microarray is a powerful tool for analysis of genetic characteristics of circulating and emerging influenza viruses and can be used to track movement of known variants. Although only 1 type of M gene (H1N1), which is relatively conserved among influenza A viruses, was tiled on RPM version 1, it was still able to cross-hybridize and differentiate M genes from different subtypes (Figure 1 and Table 4). This tiled gene would theoretically allow detection of any other type of influenza virus for which antigenic HA and NA sequences were not tiled on the array.

Another powerful feature of RPM version 1 is its broad-spectrum detection capability, allowing simultaneous resequencing of dozens of gene targets from multiple pathogens in 1 assay. This capability, however, is dependent on an equally broad-spectrum amplification method. With 66 diverse gene probes tiled on RPM version 1 covering 20 common respiratory and 6 biothreat pathogens (*14*), it was logical to use a generic, sequence-independent PCR strategy to amplify all potentially pathogen-derived sequences in an unbiased fashion before hybridization. By adopting a random amplification protocol (*18*) for use with RPM version 1, we could simultaneously detect multiple microorganisms, as shown with trivalent FluMist vaccine.

Correctly identifying 4 different influenza subtypes and their corresponding genes provided a simultaneous demonstration of 3 features of the resequencing microarray: strain identification through pattern recognition, sequence determination, and broad-spectrum capability. Conventional sequencing can determine DNA sequence and has been routinely used for genetic typing in surveillance investigations (*16**,**17**,**21**,**29*). However, it requires designing specific primers and multiple RT-PCRs to determine and amplify individual genes (such as HA, NA and M) before proceeding with sequencing reactions (this is especially true for highly polymorphic RNA viruses such as influenza virus). This requires initial use of other lower resolution techniques to identify strain type. All of these steps are time-consuming and labor-intensive. RPM version 1, combined with a random amplification protocol, can provide sequence information about a wide variety of genes representing many pathogens simultaneously and rapidly without knowledge of the identity of the tested sample. With the current possibility of an avian influenza virus A/H5N1 pandemic (*30*), surveillance for and characterization of emerging variants are essential to the rapid implementation of control measures.

In conclusion, we have combined a random amplification strategy with a resequencing microarray to efficiently and simultaneously detect, type, and genetically characterize geographically diverse influenza viruses. Application of this and similar methods may aid in a better understanding of the incidence, prevalence, and epidemiology of influenza infections and simultaneously allow more rapid identification of epidemic and pandemic outbreaks.
